# Supplementary figures and images for: Individual differences in self-reported lie detection abilities
Source: PLoS One. 2023 May 24;18(5):e0285124. doi: 10.1371/journal.pone.0285124 (PMC10208523; doi:10.1371/journal.pone.0285124)

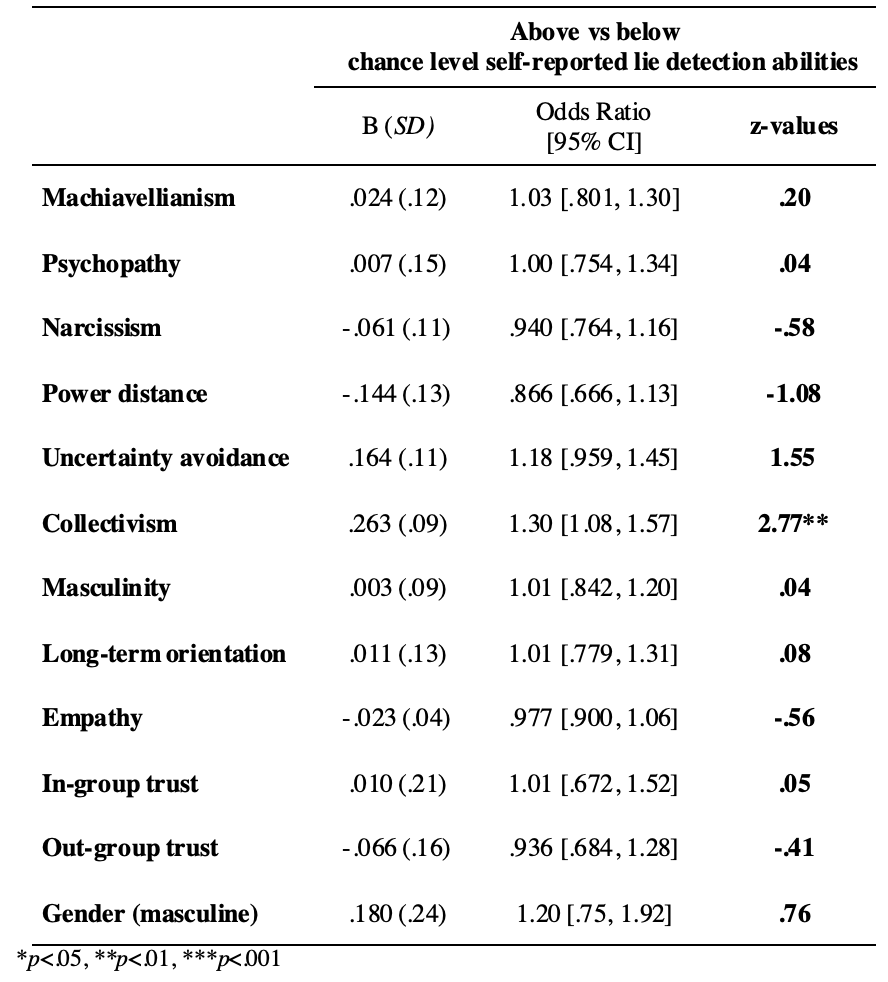

Supplement: S1 Table — (TIF) [file pone.0285124.s001.tif]

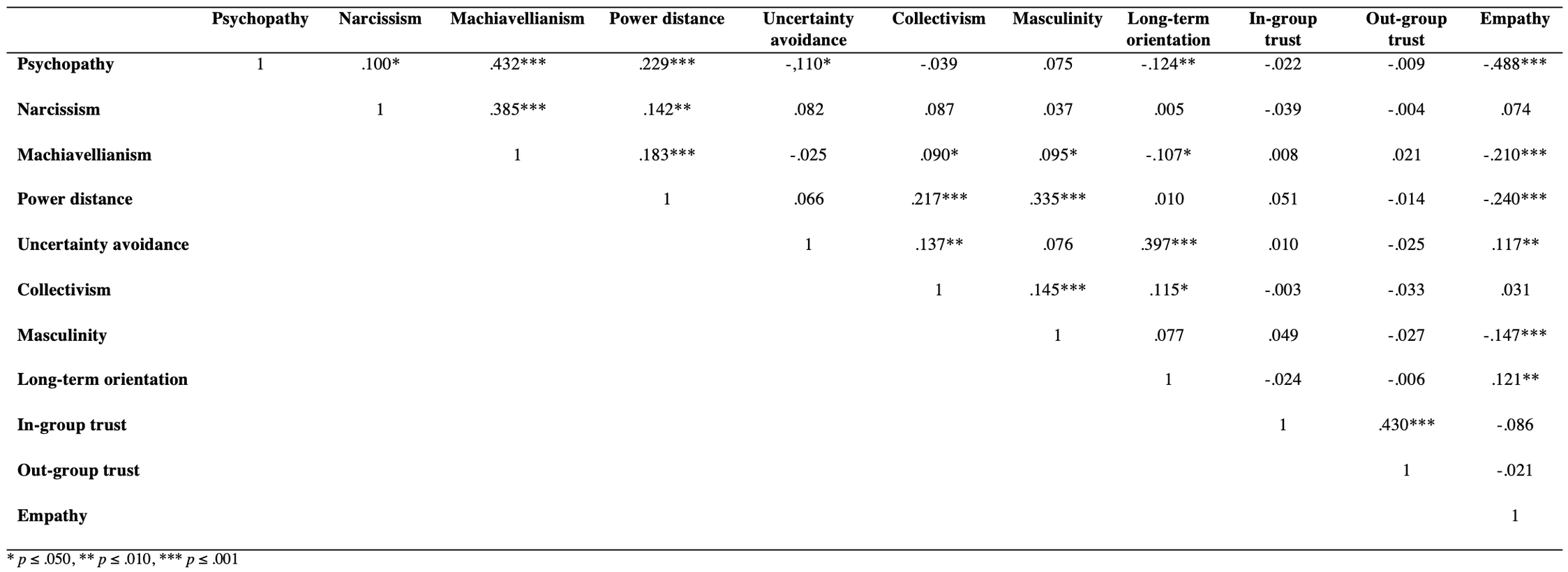

Supplement: S2 Table — (TIF) [file pone.0285124.s002.tif]

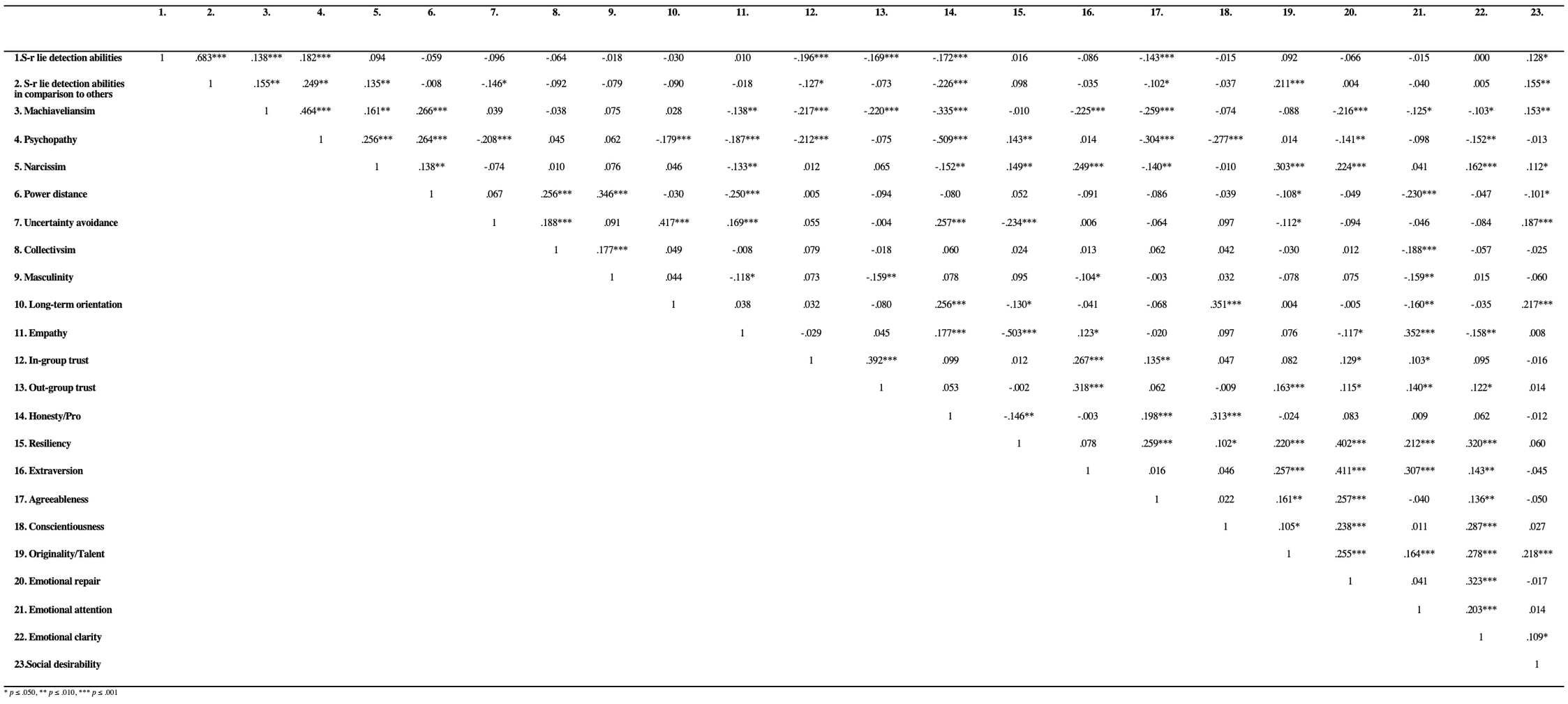

Supplement: S3 Table — (TIF) [file pone.0285124.s003.tif]
